# Management of patients with uncontrolled arterial hypertension – the role of electronic compliance monitoring, 24-h ambulatory blood pressure monitoring and Candesartan/HCT

**DOI:** 10.1186/1471-2261-6-36

**Published:** 2006-08-30

**Authors:** Thomas Mengden, Hans Vetter, Eric Tousset, Sakir Uen

**Affiliations:** 1 Internal Medicine, University of Bonn, Bonn, Germany; 2 AARDEX Ltd., Visé, Belgium

## Abstract

**Background:**

Incomplete drug regimen compliance (DRC) and white-coat hypertension are two of several possible causes of uncontrolled hypertension. Therefore the aim of the present study was to compare DRC in hypertensives treated with combination therapy whose blood pressures (BP) were controlled vers. uncontrolled after 4 weeks of self-monitored BP measurement. To observe the consequences in uncontrolled patients of switching one drug of the combination therapy to candesartan/HCTZ (16 mg/12.5 mg) with and without a compliance intervention program.

**Methods:**

Self-and ambulatory-monitoring of BP were done with upper arm oscillometric devices. Patients' dosing histories were compiled electronically (MEMS(c), AARDEX). Patients with office blood pressure (OBP) >140/90 mmHg despite combination therapy were begun on MEMS monitoring and self BP measurement for 4 weeks of run-in. Of 62 such patients, 18 (29%) patients were normotensive according to self BP measurement and ambulatory BP measurement at 4 weeks (Group A); in the remaining 44 still uncontrolled patients, candesartan/HCTZ was substituted for one of the combination therapy drugs, with half these patients receiving passive compliance monitoring (B) and half a DRC intervention program (C). All groups were then followed for 8 weeks.

**Results:**

DRC before week 4 was significantly higher in A than in the uncontrolled patients (B&C). DRC was stable during run-in A, but declined in B and C. DRC after week 4 was not different in the three groups and stayed constant over time. DRC during weekends was lower than during weekdays in all groups.

In group A no significant change in blood pressure was observed with all three methods of BP measurements. In groups B and C significant reductions of systolic and diastolic BP were observed for ABPM and SBPM. After the change to candesartan/HCTZ in B&C ambulatory 24-h-BP (ABPM) was normalized in 39% of patients.

**Conclusion:**

Normalization of BP was associated with superior drug regimen compliance in previously uncontrolled patients treated with a combination drug regimen. Switching still-uncontrolled patients to candesartan/HCTZ significantly improved BP control and stabilized a declining DRC.

## Background

Understanding and optimizing the management of hypertension is complicated by the lability of blood pressure, the sources of which include (a) white-coat effects, (b) variable exposure to prescribed antihypertensive drug(s), created by patients' variable but infrequently measured compliance with prescribed dosing regimen(s), (c) different magnitudes and time-courses of action of the many antihypertensive drugs, each of which has its own characteristic pharmacokinetic and pharmacodynamic behavior, (d) diurnal fluctuations in blood pressure, which may exceed in magnitude the hypotensive effects of antihypertensive drugs.

A further disappointing finding has been pharmaco-epidemiologic data showing a high proportion of treated patients who discontinue treatment for no clear reason within some months or a year or two after the start of treatment [1,2]. This problem being called 'short persistence' in the pharmaco-epidemiologic literature. Thus, we have the twin problems of too few patients effectively treated for too short a time.

Candesartan cilexitil (Candesartan) is a highly selective and long acting AT1 receptor blocker with excellent blood-pressure lowering efficacy and a prolonged duration of antihypertensive efficacy exceeding 24 hours. In a "missed dose" study by Lacourcière and Asmar the duration of action of candesartan suggested that this AT1 receptor blocker may provide added confidence of therapeutic coverage in many patients who may not be 100% compliant with their once-daily medication regimen [3].

The realization of such pharmaceuticals has, however, not solved either the compliance or the persistence problems [4]. We have good tools, but poor methods for applying them in long-running programs of patient management.

Against that background, the present study was designed as a pilot study in a group of longtime treatment-resistant hypertensives, to explore some aspects of the respective roles of poor compliance with prescribed drug regimens, persistent unresponsiveness to correctly administered drug, and several modest interventions – a change in one of the prescribed drugs, use of ambulatory blood pressure monitoring, informing patients that their dosing histories were being continuously compiled, and some feedback to patients of both blood pressure data and dosing history data.

## Methods

**Figure 1:**
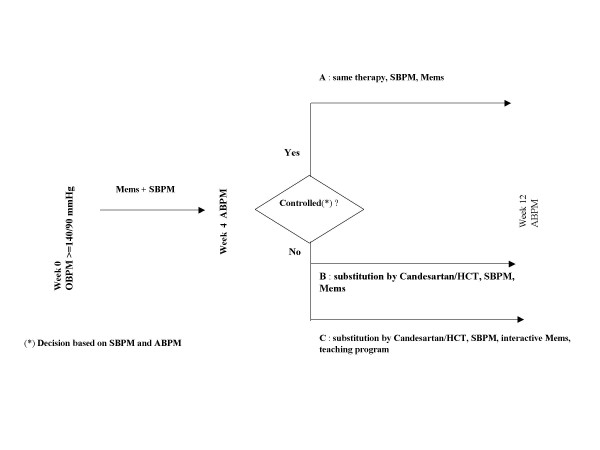
Design of the study.

Figure 1 outlines the design of the study. Patients with essential hypertension and office blood pressure (OBP) ≥ 140/90 mmHg, despite combination therapy (> 2 antihypertensive drugs), were monitored by MEMS (Medication event monitoring system, Aardex company Switzerland) and by self-measurement of blood pressure (SBPM) during a 4 week period. In patients with elevated SBPM and 24-h ambulatory blood pressure (ABPM) after the run-in period, one drug of the hypertensive regimen was substituted by candesartan/hydrochlorthiazide (16/12.5 mg). Patients with normotension after run-in were judged to have a white-coat effect, and were assigned to group A as a comparison-group for the remainder of the patients, who were persistently hypertensive and were randomized to groups B or C. Group C received a structured hypertension teaching program [5] and interactive MEMS Monitor with a visiual reminder of the number of drugs taken and the time intreval elapsed since the last drug intake. We used an informal, shortened teaching program (30 min.), which included the following items and was always performed by the same investigator (S.U.)

a. Definition of normal values for office, SBPM and ABPM and target BPs

b. Prognostic implication of uncontrolled hypertension

c. Effect of non-compliance on control rate

d. Correct use of SBPM

In contrast, Group B was managed in the usual manner, with dosing history data being compiled by a standard, non-interactive MEMS Monitor, but without any special effort being made to improve the patients' compliance.

Patients were consecutively recruited from the outpatient department of the Medizinische Poliklinik. Patients were refered to our department by GPs for resistant or difficult to treat hypertension. All patients gave informed consent to participate in the study and were informed about the function of the MEMS system. The University of Bonn issued a positive ethical vote on the study.

### MEMS system

Candersartan was dispensed in Medication Event Monitoring System (MEMS^®^, AARDEX Ltd.) containers. The MEMS system comprises an electronic pill-bottle cap embedding a microship that creates an electronic time-stamp upon opening and closing the bottle. These time-stamps comprise a complete dosage history. Mems caps may also include a LCD display that reminds the patient the number of pills taken since the beginning of the day. In this article, the term interactive MEMS refers to the devices with screen displays. The recorded data can then be downloaded to a personal computer using Powerview software (Aardex Ltd.). This software offers features to visualise and compute basic individual statistics. Adherence to Candersartan was assessed using MEMS devices over the course of the study. Both interactive and non-interactive devices were used depending on the intervention group.

**Figure 2:**
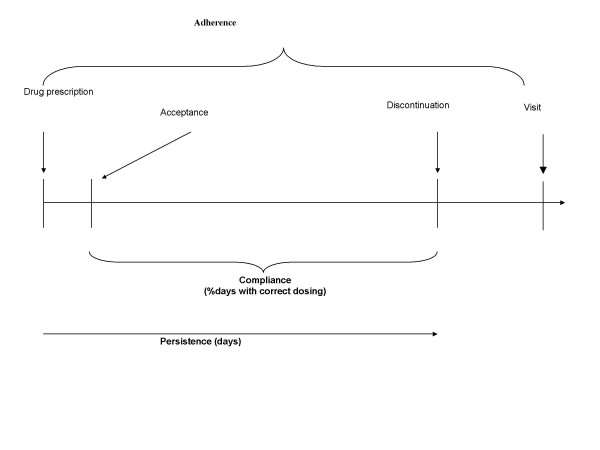
Formal definition of adherence/compliance/persistence.

The quality of execution of the prescribed drug regimen is called 'compliance', defined as 'the extent to which the patient's dosing history corresponds to the prescribed regimen' (Fig. 2). The length of time between onset and discontinuation of execution is called 'persistence'. The general term, 'adherence' subsumes acceptance, execution, and discontinuation. 'Poor adherence signals that something is or was wrong with the drug regimen – not accepted, or badly executed and/or discontinued prematurely. In this context, compliance will be measured by the percentage of days with correct intake (*cod*) and the percentage of prescribed number of doses taken (*tac*).

### Blood pressure monitoring

Self measurement was performed with a validated, oscillometric upper arm device, A&D UA-767 [7], After sufficient training in the correct method of blood pressure measurement, duplicate measurements were performed between 6–9 AM (before medication) and 6–9 PM for at least three days/week for the whole study period. Data were stored electronically and were downloaded to a PC at the end of the study.

Ambulatory 24-h BP measurement was done with the "CardioTens-01" (Meditech, Budapest, Hungary) device [6], which measured blood pressure oscillometrically every 15 minutes in the period between 10.00 a.m. and 10.00 p.m., every 30 minutes between 10.00 p.m. and 06.00 a.m. and once every 10 minutes between 06.00 a.m. and 10.00 a.m. This method of blood pressure monitoring has been validated in accordance with the protocol of the British Hypertension Society.

The definitions of controlled hypertension are: self-measurement <135/85 mmHg, office <140/90 mmHg, ABPM (whole 24 h period) <130/80 mmHg. We decided to work on ABPM since this technique has important prognostic value, especially in patients with refractory hypertension [8]. Patients with discrepant results of ABPM and SBPM at week 4 were excluded from the analysis (2 patients).

### Statistical methods

The non-parametric Wilcoxon test was used to compare adherence distribution between groups. Comparison of binary time series were realized using a logistic regression where dependence among observations from a same patient over time was taken into account through a conditional approach. Comparisons of BP values were performed with the t-test for comparison between baseline and week 12. All significance tests are two-sided and statistical significance is set at 5%. All analyses are performed using the S-plus^® ^version 6.2. BP values are expressed as means+S.D. In case of multiple comparisons between groups, a Bonferroni's correction of the significance level has been applied.

Sample size was calculated to detect a 5 mmHg difference in systolic ABPM at 90% power and p < 0.05 in 20 patients.

## Results

Sixty-two patients with essential hypertension and OBP ≥ 140/90 mmHg, despite combination therapy, were enrolled (26 female and 36 male, age 61+9 years). After 4 weeks of run-in, 18 of these (29%) were normotensive at 4 weeks, and, on that basis, were designated Group A. For the remaining 44 evaluable, still-uncontrolled patients, candesartan/hydrochlorothiazide was substituted at week 4 for one of the previously prescribed combination therapy drugs. The choice of drug was left to the discretion of a experienced hypertension specialist. These 44 patients were randomized between group B (n = 20), to continue receiving passive compliance monitoring, and Group C (n = 24), to receive an intervention program designed to improve drug regimen compliance. Group B had 20 evaluable patients and C had 24. All groups were then followed for 8 weeks. The characteristics of patients with different antihypertensive drugs used at the beginning of the study are presented in table 1. There were no significant differences between groups B and C. Patients of group B and C had more diabetes than patients of group A. The classes of antihypertensive drugs which were replaced by candesartan/HCTZ in groups B and C patients after the run-in period are given in table 2.

**Table 1:** Baseline characteristics of patients

Groups	A	B	C
Age (y)	58 ± 10	60 ± 10	64 ± 6
Sex (male)	9	14	14
Height (cm)	169 ± 8	172 ± 9	170 ± 8
Weight (kg)	78 ± 12	85 ± 14	79 ± 15
BMI (kg/m^2^)	27 ± 3.2	29 ± 4.1	27 ± 3.7
Coronay heart disease	7	12	10
Myocardial infarction	3	3	2
Diabetes	0	10	8
Smoking	4	0	4
Dyslipidemia	12	10	15
Beta-blocker	13	11	16
Diuretics	13	15	13
Calcium channel blocker	6	10	11
ACE-inhibitor	13	11	17
Alpha 1-Receptor-blocker	0	5	3
Angiotensin Receptor Blocker	3	3	4

**Table 2:** Classes of antihypertensive drugs replaced by candesartan/HCT after run-in period

Groups	B	C
Diuretics	10	11
ACE-inhibitor	5	6
Calcium channel Blocker	2	2
Angiotensin Receptor Blocker	3	4
Alfa 1-Receptor-blocker	0	1

### Compliance

#### Summarizing the history in an overall measure

We consider simple statistics to summarize the patient's compliance history. The statistics used in this context are the percentage of days with correct intake (*cod*) and the percentage of prescribed number of doses taken (*tac*). Those quantities were computed for each patient during the two phases of the study. Table 3 summarizes the different compliance statistics during the two phases in the different groups. Using a Wilcoxon test to compare compliance statistics between the randomized groups, none of the measures discussed shows a significant difference between randomized groups at the nominal 5% level. Although the measures provide a well-understood, simple description, patient compliance can vary in many different ways over time. Summarizing the history in just a few measures may lose power to detect relevant differences in compliance patterns. The next logical step is to capture the temporal evolution of the daily compliance. The next sections describe relevant methodology.

**Table 3:** Compliance summary statistics

Groups	**Week 0–4 (Adherence)**		**Week 4–12 (Compliance)**	
	
	Cod (*)	Tac (*)	Cod (*)	Tac (*)
	15 days preceding the Week 4 visit	15 days preceding the Week 4 visit	Between the first and last events occurring between weeks 4 and 12	Between the first and last events occurring between weeks 4 and 12
**A**	Median 0.9667 Mean 0.8625	Median 1.0000 Mean 0.9563	Median 0.9652 Mean 0.9133	Median 1.0045 Mean 0.9775
**B**	Median 0.9333 Mean 0.7770	Median 1.0000 Mean 0.9046	Median 0.9815 Mean 0.9281	Median 1.0000 Mean 0.9770
**C**	Median 0.9333 Mean 0.7770	Median 1.0000 Mean 0.9046	Median 1.0000 Mean 0.9709	Median 1.0000 Mean 0.9973

(*) cod = correct dosing.

tac = taking compliance.

#### Analysing adherence over time before week 4

**Figure 3:**
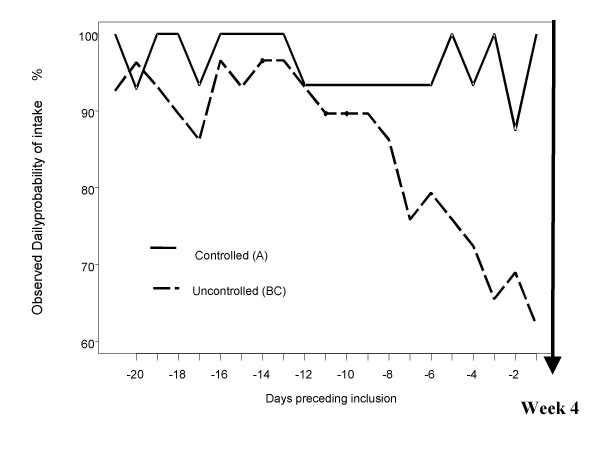
Observed Daily probability of intake preceding the week 4 visit. Difference between the adherence in the controlled patients and the non controlled patients: the adherence of non controlled patients was significant  lower and the decrease steeper.

The aim of this section is to describe the evolution of an informative measure of adherence in randomized treatment groups. Because adherence to prescribed therapy measures the extent to which a person's behavior coincides with medical advice, it covers both a behavior and a measure. One factor influencing the other delivers raw data that have a complex structure with unusual correlation patterns of dosing intervals, and it is not obvious how to analyze these patterns. Rather than study detailed patterns, we summarize the adherence pattern in a sequence of binary data *Z*_*ij *_indicating whether yes (1) or no (0), at least the prescribed number of doses were taken on day *j *by patient *i*. This coding retains much of the temporal structure in the individual patterns, but not exact times of drug doses [9]. The observed proportion of good adherers on each separate day before week 4 visit, i.e., patients having taken at least their prescribed number of doses, is presented in Figure 3. The adherence in group BC has been found significantly lower than in group A (p < 0.001). Finally the model has confirmed a significant decrease of adherence over time in group BC (p = 0.012).

#### Analysing compliance over time after week 4

**Figure 4:**
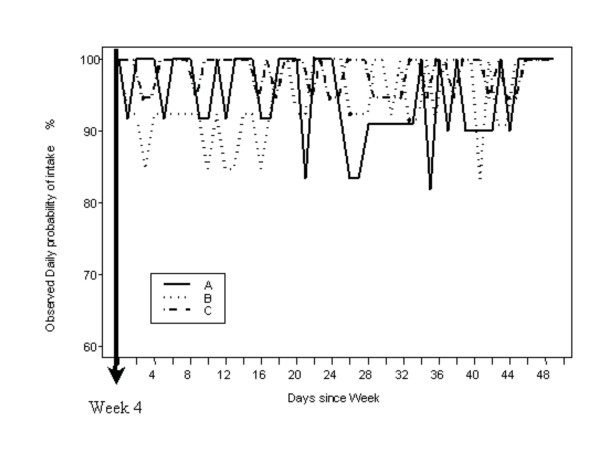
Observed Daily probability of intake after week 4 visit. The compliance after week 4 is not different in the three groups and stayed constant over time.

The observed proportion of good compliers on each separate day after week 4 visit is presented in Figure 4. The resulting model did not highlight any significant differences between the three groups. A significant decrease over time could not be identified. Interestingly, it showed the existence of a weekend effect leading to a lower daily probability of intake during the weekend defined as Friday, Saturday and Sunday. The odds ratio of taking a dose the weekend (Friday – Sunday) compared to the odds of taking the rest of the week was 50.1 % (95% confidence interval : [28.1–90.0]).

#### OBP, SBPM and ABPM blood pressure control

Table 4 shows the blood pressure values for all three methods of measurement after the run-in period (visit week 4).

**Table 4:** Blood pressure values for all three methods of measurement after the run-in period (visit week 4).

	**Group A Week 4**	**Group B Week 4**	**Group C Week 4**
**BP method**			
**OBPM syst**	150 ± 17 #	162 ± 15	161 ± 16 n.s.
**OBPM diast**	88 ± 10	89 ± 13	88 ± 13 n.s.
**SBPM syst**	128 ± 5 #	155 ± 10	154 ± 12 n.s.
**SBPM diast**	79 ± 5 #	84 ± 12	84 ± 12 n.s.
**ABPM syst**	122 ± 6 #	149 ± 10	144 ± 11 n.s.
**ABPM diast**	74 ± 5 #	80 ± 12	78 ± 10 n.s.

OBPM: Office blood pressure measurement.

SBPM: Self blood pressure measurement.

ABPM: Ambulatory 24-h blood pressure measurement.

syst = systolic, diast = diastolic.

#: t-test p < 0.05/3 (Bonferroni's correction for multiple testing A vs B, A vs C and B vs C).

n.s. = B vs C.

**Figure 5:**
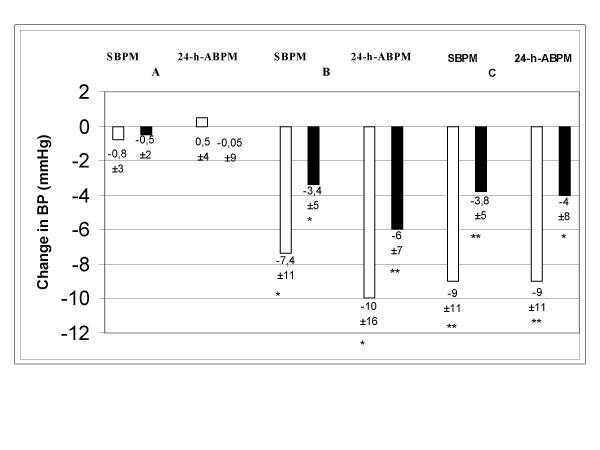
Change in blood pressure between visit week 4 and visit week 12. For 24-h-ABPM the respective values of the last day of run-in and the last day of week 12 are compared. For SBPM the mean values 14 days preceding week 4 visit are compared with the 14 days preceding week 12 visit are compared. White bars: change of systolic blood pressure value, black bars: change of diastolic blood pressure value. *: t-test p < 0.05. **:t-test p < 0,01.

The change in blood pressure between visit week 4 and visit week 12 is shown in the figure 5.

For SBPM the mean values 14 days preceding week 4 visit are compared with the 14 days preceding week 12 visit are compared. For ABPM the respective values of the last day of run-in and the last day of week 12 are compared. As expected in group A with unchanged medication no significant change in blood pressure was observed with all three methods. In groups B and C significant reductions of systolic and diastolic BP were observed for SBPM and ABPM.

The blood pressure lowering effect of substitution by Candesartan/HCT was most pronounced as assessed by systolic OBPM (162 ± 15 vs 148 ± 17 for group B and 161 ± 16 vs 142 ± 23 for group C; p < 0.025) whereas diastolic OBPM reduction did not change significantly.

The BP lowering effect resulted in normlization of ABPM in 39 % of patients in group B and 39% of patients in groups C. The confounding created by white-coat effect is illustrated by the data from Group A: at week 12, 80% were uncontrolled by OBP, whereas only 17% were uncontrolled as assessed by ABPM.

At the end of the study 53% of all patients included (group A, B, and C) had normal ABPM values, i.e. <130/80 mmHg for the whole 24-h period.

#### Number of antihypertensive drugs

The average number of antihypertensive drugs before intervention (week 4) were 2.8+0.6, 2.8+0.9 and 2.8+0.9 for groups A, B and C respectively. The mean number of drugs after intervention significantly increased to 3.7+0.8 and 3.6+0.9 in groups B and C respectively.

## Discussion

An overall result of this study was that the various maneuvers executed between the start and end of the study resulted in the normalization of blood pressure in 53% of the patients who had been persistently hypertensive by OBP measurement despite treatment with multiple drugs. The various maneuvers included: start of both home blood pressure monitoring and drug regimen compliance monitoring at week 0, exclusion of uncontrolled hypertension caused by a white-coat effect, change of the drug regimen in Groups B and C at week 4, and the commencement of a compliance-enhancement program in Group C at week 4. In the persistently hypertensive groups B and C, 39% achieved normalization of blood pressure. In the present study, a number of factors made it impossible to deduce which of the various maneuvers, including compliance-improvement, played the dominant role in the normalization of blood pressure, nor is it evident what sorts of reductions in the number of drugs or doses prescribed might be possible in correctly dosing former poor compliers, without loss of control over blood pressure.

A fortunate aspect of the present study is that the maneuvers performed were fairly simple and cost effective, and without discernible hazards, so that repetition with better collection of data and simplified designs may allow identification of the most important maneuvers.

Compliance in week 0–4 was monitored with up to 3 MEMS boxes in individual patients. Since – as in previous studies – no difference could be observed between 1 or more MEMS boxes, we decided to continue on 1 MEMS box for Cande/TZD

During the run-in period, the percentage of prescribed doses taken, which is one of several ways to express drug regimen compliance, fluctuated to a slight extent, but remained essentially constant in Group A. In contrast, the patients who were destined to comprise Groups B and C showed a more or less continuous decline in percentage of prescribed doses taken, from the mid-point to the end of the 4-week run-in period, during which time the mean percentage of prescribed doses taken declined from 93% to 62%.

Remarkably, compliance in Groups B and C improved immediately following the visit at week 4 and continued, as shown in Figure 4, to fluctuate from day to day in the region between 85% and 100% of prescribed doses taken. A possible explanation of this phenomena is the switch to a more efficient therapy and a QD regimen.

A notable finding was a substantially higher incidence of omitted doses on weekends (Fridays-Saturdays-Sundays) than on weekdays (Mondays thru Thursdays). This phenomenon was also reported in a previous work by Mallion et al. [10]. This prominent effect does not show up in Figure 4, as the data are plotted on an ordinal day basis, rather than a calendar day basis – an object lesson in how some methods of displaying averaged time-series data can camouflage important features.

Following the change to candesartan/HCTZ in Groups B&C significant BP reductions could be observed with all three methods of BP monitoring. Since ABPM is more useful than OBPM in stratifying cardiovascular risk in patients with refractory hypertension, we used the normalization criteria of ABPM. It is remarkable that a normalization rate of 39% was achieved as monitored by ABPM in groups B&C. This beneficial finding is in keeping with results of prior studies, and may in part be due to the relatively long duration of action of candesartan, which could be expected to have the effect of allowing antihypertensive action to persist in the face of delayed or omitted doses [11-14]. The better blood pressure control at week 12 may be explained by both improved compliance and switch to Candesartan/TZD. However, our study design did not allow to give a good estimate of the relative contribution of each factor. But Burnier et al could demontrated in a prior study that monitoring of compliance alone was associated with a significant improvement of blood pressure at 2 months (systolic/diastolic blood pressure:156/106 +/- 23/11 versus 145/97 +/- 20/15 mmHg, P < 0.01) [15].

Drug regimen compliance after week 4 was not different in the three groups, staying essentially constant over time, very close to correct dosing each day, with of course the exception of the 'weekend effect', the pressure correlates of which we did not explore. Relatively long-acting antihypertensive agents, e.g., candesartan, can 'forgive' dose omissions occurring on weekends, whereas relatively short-acting drugs, that cannot maintain pressure control for at least 48 hrs after a last-taken dose, cannot [11].

### Limitations of the study

Clearly the transactions between investigative staff and patients had substantial though variably sustained impact. We do not know – and it is an important omission that should be avoided in future studies – what the dosing histories and thus the compliance levels were in the period prior to the start of the run-in period. One naturally wonders if it were not generally poor in many or most of the patients before the start of the study, whereupon the first measurements showed it to be high in all patients, perhaps a forerunner of the abrupt jump in compliance that we see in Groups B and C immediately after the week 4 visit. Perhaps – but we can only speculate and take the point in designing future studies – the patients who were to comprise Group A were, prior to the start of the study, poorly compliant, but received enough motivation from the various maneuvers made at the start of the study, so that their compliance rose and their blood pressures concomitantly normalized, where they remained throughout the 12-week study. A high priority, therefore, is to master – as some investigators have [16,17] – the art of introducing electronic monitoring in an unperturbing manner, so that valid pre-study dosing histories can be captured.

For reasons that this study does not reveal, the week 4 visit restored average compliance in Groups B and C to the same high range it had in the first 2 weeks after of the initial visit at the start of the study. One naturally wonders whether maneuvers begun at the start of the study, 4 weeks previously, had not had a similar effect, akin to that which we see after week 4, of improving a previously unsatisfactory compliance. It is also a matter of conjecture about which of the various maneuvers uniquely associated with the conduct of the study played the biggest role in (a) achieving a sustained increase in compliance in Groups B and C, and (b) achieving blood pressure control in a fraction of the patients in Groups B and C.

Another limitation of the study was the rather short duration of the study. It is well known that long term, "real life " compliance may not be reflected by short observation periods 1 or 2 months.

## Conclusion

This study demonstrated blood pressure normalization in a 53% of previously uncontroled hypertensives. Clearly, the use of ABPM and SBPM were helpful in identifying white-coat effects. The mechanisms for this improvement are not clear, but each of the maneuvers that preceded this improvement are simple, safe, easily applied, and relatively economical. Further work, taking cognizance of the shortcomings of the present study, can probably differentiate the more from the less effective of the maneuvers, and help improve the proficiency of their use.

## Abbreviations

OBP: Office blood pressure

SBPM: Self blood pressure measurement

ABPM: Ambulatory 24-h blood pressure measurement

MEMS: Medication Event Monitoring System

Interactive Mems: Mems with screen display

## Competing interests

This study was supported by AstraZeneca Germany.

Eric Tousset is an employee of Aardex, the company manufacturing the monitors used in this study.

All other authors declare that they have no competing interests.

## Authors' contributions

TM conceived of the study, and participated in its design and coordination and drafted the manuscript. ET performed the statistical analysis of compliance and blood pressure parameters. VH participated in the design of the study and coordination. SU recruited the patients, supplied blood pressure measurements and electronic pill boxes, hold the structured hypertension teaching program and drafted the manuscript. All authors read and approved the final manuscript.

## Pre-publication history

The pre-publication history for this paper can be accessed here:


